# Silver Nanowire Networks: Ways to Enhance Their Physical Properties and Stability

**DOI:** 10.3390/nano11112785

**Published:** 2021-10-21

**Authors:** Laetitia Bardet, Dorina T. Papanastasiou, Chiara Crivello, Masoud Akbari, João Resende, Abderrahime Sekkat, Camilo Sanchez-Velasquez, Laetitia Rapenne, Carmen Jiménez, David Muñoz-Rojas, Aurore Denneulin, Daniel Bellet

**Affiliations:** 1 Univ. Grenoble Alpes, CNRS, Grenoble INP, LGP2, F-38000 Grenoble, France; aurore.denneulin@grenoble-inp.fr; 2 Univ. Grenoble Alpes, CNRS, Grenoble INP, LMGP, F-38000 Grenoble, France; theodora.papanastasiou@grenoble-inp.fr (D.T.P.); chiara.crivello@grenoble-inp.fr (C.C.); masoud.akbari@grenoble-inp.fr (M.A.); abderrahime.sekkat@grenoble-inp.fr (A.S.); camilo.sanchez-velasquez@grenoble-inp.fr (C.S.-V.); laetitia.rapenne@grenoble-inp.fr (L.R.); carmen.jimenez@grenoble-inp.fr (C.J.); david.munoz-rojas@grenoble-inp.fr (D.M.-R.); 3 AlmaScience Colab, Madan Parque, 2829-516 Caparica, Portugal; joao.resende@almascience.pt

**Keywords:** transparent electrode, percolation, optimization, stability, conformal coating, spatial atomic layer deposition, metallic nanowire, figure of merit, nanocomposite, thermal treatment

## Abstract

Silver nanowire (AgNW) networks have been intensively investigated in recent years. Thanks to their attractive physical properties in terms of optical transparency and electrical conductivity, as well as their mechanical performance, AgNW networks are promising transparent electrodes (TE) for several devices, such as solar cells, transparent heaters, touch screens or light-emitting devices. However, morphological instabilities, low adhesion to the substrate, surface roughness and ageing issues may limit their broader use and need to be tackled for a successful performance and long working lifetime. The aim of the present work is to highlight efficient strategies to optimize the physical properties of AgNW networks. In order to situate our work in relation to existing literature, we briefly reported recent studies which investigated physical properties of AgNW networks. First, we investigated the optimization of optical transparency and electrical conductivity by comparing two types of AgNWs with different morphologies, including PVP layer and AgNW dimensions. In addition, their response to thermal treatment was deeply investigated. Then, zinc oxide (ZnO) and tin oxide (SnO_2_) protective films deposited by Atmospheric Pressure Spatial Atomic Layer Deposition (AP-SALD) were compared for one type of AgNW. We clearly demonstrated that coating AgNW networks with these thin oxide layers is an efficient approach to enhance the morphological stability of AgNWs when subjected to thermal stress. Finally, we discussed the main future challenges linked with AgNW networks optimization processes.

## 1. Introduction

Metallic nanowires (MNWs) started to be investigated after significant progress in the synthesis of silver nanowires (AgNWs) [1], followed by copper nanowires (CuNWs) [2], as well as copper-nickel nanowires (Cu–Ni NWs) [3] or other bimetallic nanowires [4]. The random networks formed by MNWs show excellent properties in terms of optical transparency and electrical conductivity [5,6]; therefore, they exhibit great potential as transparent electrodes (TE) [7,8] integrated within many devices, such as solar cells [9,10], transparent heaters [11,12,13], organic light-emitting diodes [14], smart windows [15], flexible pressure sensors [16], supercapacitors [17] or touch screens [18]. Moreover, MNW networks have demonstrated promising assets for several applications, not necessarily related to TE, such as antimicrobial activity [19], water purification [20], memristive devices [21] or low-emissivity films [22]. Regardless of the targeted application, it is crucial to optimize the physical properties of MNW networks to ensure an efficient integration within devices. Nevertheless, there is no consensus-based global optimization method, since this issue depends highly on the target application, with its specific features and constraints, but it is also dependent on the chemical nature and dimensions of the MNWs themselves.

The main physical properties to be optimized are optical transmittance (*T_r_*) and sheet resistance (*R_sh_*); usually, TE require optical transmittance to be above 90%, with sheet resistance around 10–20 Ω/sq. One way to assess this performance is to use a figure of merit (*FoM*), the most traditional being the one proposed by Haacke in 1976, as follows [23]:

(1) $$\;\;FoM^{Haacke}=\frac{T_{r}{}^{10}}{R_{sh}}$$

Another *FoM* is also often used in literature, since it does not appear to be connected to any particular transparency value: it considers the ratio between the electrical conductivity, σ, and the optical conductivity, which is defined as the optical absorption coefficient, *α*, divided by the vacuum impedance, Z_0_ [24]. However, these *FoMs* do not consider other properties or features that are important to assess the performance of TE, depending on the targeted device; for instance, these include haziness (ratio between diffuse and total transmittance), mechanical properties, thermal stability, etc. 

The present article focuses on AgNW networks, which are the most promising MNW candidates to replace Indium Tin Oxide (ITO), which is the most used so far in industry. ITO is brittle and not very transparent in the near-infrared (NIR) region, while AgNW networks appear very flexible [5] and well transparent in the NIR region [25]. Other metallic nanowires can also been considered: for instance, CuNWs exhibit appealing assets [26] due to their much lower cost and the absence of plasmonic effects. However, they do suffer from lower stability associated to rapid oxidation [27].

After a brief summary of the main properties of AgNW networks and recent ways to optimize them, we have investigated a novel way to optimize the electrical and optical properties of two types of AgNWs. These AgNWs have been synthesized by a polyol process with different synthesis conditions, leading to different morphologies and dimensions. The effects of post-deposition treatment, such as thermal annealing, have been compared for these both types of AgNWs. Then, we have studied the effects of conformal coating on AgNWs, with a specific focus on two thin oxide coatings: zinc oxide (ZnO) versus tin oxide (SnO_2_), both deposited by Atomic Pressure Spatial Atomic Layer Deposition (AP-SALD). These two oxides appear very pertinent for future use of AgNW-based TE, since they are often used within solar cells [28,29]. This work briefly ends with the main challenges and prospects related to the optimization processes of AgNW networks.

## 2. Materials and Methods

### 2.1. Details about AgNWs and Deposition Technique 

Two types of AgNW suspensions were used in this work. The first one was kindly provided by the research team of Jean-Pierre Simonato from CEA-LITEN (Grenoble, France), and it was produced according to a protocol published by Mayousse et al. [30]. The second type of AgNWs was purchased from R&Dnano (Strasbourg, France) [31]. The synthesis process of AgNWs for both types of AgNW relied on polyol synthesis in ambient air using ethyleneglycol solution as a solvent. Table 1 reports details provided by suppliers regarding their synthesis conditions and the dimensions of the AgNWs after synthesis. AgNWs were deposited on Corning^®^ glass substrates (Delta Technologies, Limited, Loveland, CO, USA) using a home-made pneumatic spray system [32]. The AgNW network area was 1.25 × 1.25 cm^2^ and 2.5 × 2.5 cm^2^ for thermal annealing studies and for optical transmission measurements, respectively. An airbrush was fixed in a mechanical setup connected with a programmable automation controller (PAC Control Basic, Opto22, Temecula, CA, USA). The airbrush moved in 2D to spray onto substrates (such as glass or PET) placed on an aluminum plate. During deposition, the spray-gun moved along a regular and periodic array in X and Y directions. Several parameters could be controlled, such as size of the surface deposition, pressure applied in the spray gun, temperature of the heating plate and number of cycles. In this study, the temperature of the aluminum plate was 110 °C, with a pressure of the deposition of 1.4 bar.

### 2.2. Deposition of Oxide Coating by AP-SALD

The oxide coatings were deposited using a homemade Atmospheric Pressure-Spatial Atomic Layer Deposition (AP-SALD) system at 200 °C [33]. The sample was at a constant distance (150 µm) from the injection head, and the experimental procedure corresponded to those published in the following references: [34,35]. The growth rate for the AP-SALD was about 0.26 nm/s for ZnO and 0.02 nm/s for SnO_2_. Silver paste was deposited on the two sides of the samples as contact electrodes. It should be noted that in Section 3.3, AgNW networks were elaborated with AgNW-1 on Corning^®^ glass and were previously thermally treated at 200 °C for 1 h in air. 

### 2.3. In Situ Resistance Measurement 

The influence of thermal treatment on the electrical resistance of both AgNW types was investigated by heating the samples on a hot plate from room temperature to 400 °C in air, with a heating rate of 5 °C/min, and measuring in situ electrical resistance, with a two-probe measurement setup using a Keithley 2400 instrument (Keithley Instruments Inc., Cleveland, OH, USA), while applying a voltage of 100 mV. The setup was controlled by a LabVIEW software (National instruments, Austin, TX, USA). 

### 2.4. Characterization of AgNWs and AgNW Networks

The samples’ optical transparency was characterized by a Perkin Elmer Lambda 950 UV-Visible-NIR spectrophotometer (PerkinElmer, Waltham, MA, USA) with an integrating sphere in order to measure their total transmittance from 250 to 2500 nm. When “without substrate contribution” has been mentioned in this work, it means that the total transmittance has been divided by the value of the glass substrate.

The AgNW morphologies were analyzed with a field-emission gun scanning electron microscopy (FEG-SEM Zeiss Gemini 300, Carl Zeiss Microscopy GmbH, Oberkochen, Germany), using an accelerating voltage of 3 keV. The areal mass density (amd), which corresponds to the silver mass per unit of the surface, was estimated with the “Ridge detection” plugin of the ImageJ computer program (NIH, Bethesda, MD, USA). Ten SEM images were captured in order to get an average value of the amd for each studied network. Transmission electron microscopy (TEM) images were obtained with a JEM-2010 TEM (JEOL Ltd., Tokyo, Japan), operating at 200 kV, with a resolution of 0.19 nm. AgNWs with ZnO or SnO_2_ coatings were directly deposited on carbon-coated copper grids for TEM observations. The thicknesses of the ZnO and SnO_2_ coatings surrounding the AgNWs was measured using 10 TEM images for each oxide.

## 3. Results and Discussion

### 3.1. Brief Overview of Main Properties of AgNW Networks

To place our work in context, Table 2 briefly summarizes the main properties of AgNW networks and some studies that recently investigated ways to optimize them. 

Beyond optical transparency and electrical resistance, there are several other properties that need to be optimized for efficient integration of TE into devices, including stability, haziness, surface roughness and adhesion to the substrate.

The high electrical conductivity of silver in combination with low junction resistance (after potential help from post-deposition treatments, such as thermal treatment) leads to AgNW networks with low electrical resistance, while the empty areas between AgNWs provide conditions for high optical transparency. The dependency of electrical properties on AgNW dimensions and AgNW network density has been the subject of several studies. For instance, Lagrange et al. [37] showed that the electrical resistance of an AgNW network, *R*, with an *amd* and AgNW length, *L*, and diameter, *D*, can be written as follows:

(2) $$\;\;R\left(amd,D,\;L\right)=R_{instrum}+C\times \rho_{bulk}^{Ag}\times \left(1+\frac{\Lambda }{2D}\right)\times {\left(amd-amd_{c}\right)}^{-\gamma }$$

where $R_{instrum}\;$ is the instrumental setup resistance; $\rho_{bulk}^{Ag}$ and *Λ* are the bulk silver electrical resistivity and electrons mean free path, respectively. *amd_c_* stands for the critical *amd* and is calculated by considering only the average AgNW length [37]. γ is the percolation exponent; the value of the latter is 4/3 for a two-dimensional network [24]. *C* is an unknown constant that can be determined by fitting the experimental data with Equation (2). While the percolation theory suggests that this dependency between *R* and *amd* is only valid for *amd* values slightly above *amd_c_* [53], it appears that the agreement is still valid over much larger *amd* range values [6,37]. The prefactor $\left(1+\frac{\Lambda }{2D}\right)$ is associated with the surface scattering of electrons, as shown by Bid et al. [54].

A similar approach has been derived for optical transmittance. Since silver is a highly reflective material in the visible spectrum [55], it can be assumed that the total transmittance,$\;T_{r},$ is simply equal to unity minus the areal fraction covered by AgNWs. Following this feature, the dependency of the AgNW network,$\;T_{r}$, can then be expressed in terms of *amd*, as follows:

(3) $$\;\;\;\;\;\;T_{r}=1-\frac{4}{\pi }\times \frac{amd}{D\times \rho_{m}^{Ag}}$$

where $\rho_{m}^{Ag}$ is the bulk silver mass density. Therefore, Equation (3) predicts a linear decrease of the total transmittance versus the AgNW network *amd*, which has been confirmed experimentally as well [37,56,57]. 

### 3.2. Comparison of Optical and Electrical Properties of AgNWs with Different Synthesis Conditions

#### 3.2.1. Effects on Morphology and Optical Properties 

The intrinsic properties of AgNWs, including dimensions, morphology and surface chemistry, have a strong influence on the physical properties of AgNW networks. The latter strongly depend on the conditions of AgNW synthesis. For instance, Coskun et al. investigated different parameters that affect the final morphology of AgNWs, including temperature, injection rate, polyvinylpyrrolidone (PVP): AgNO_3_ molar ratio, NaCl concentration or stirring rate of the solution [58]. Hwang et al. demonstrated that the influence of the PVP capping layer plays a key role in the electrical resistance of the whole network. By using a washing method, they were able to change the PVP layer thickness and showed a strong influence on the contact resistance between AgNWs and, therefore, on the electrical resistance of the entire AgNW network [59]. Moreover, it could also be elevated due to the lack of close contact between AgNWs after deposition. To solve this, many investigations reported post-deposition treatments, i.e., mechanical pressing, light-induced plasmonic nano-welding, capillary-induced cold-welding or thermal annealing [39,40,41,42].

**Figure 1:**
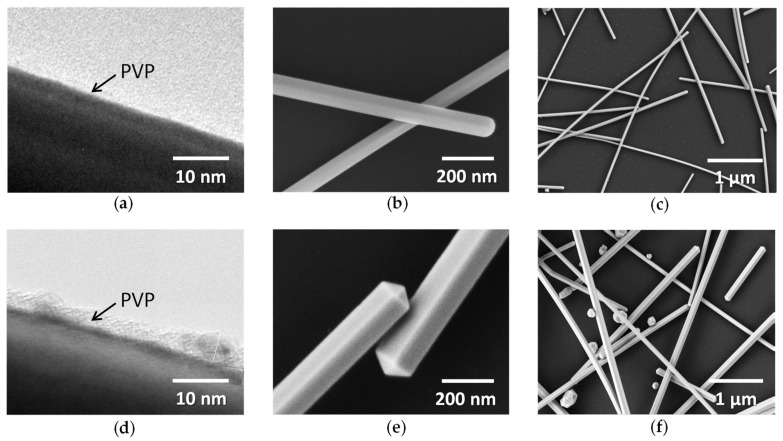
AgNWs with different morphologies and dimensions. (**a**) TEM observations and (**b**,**c**) SEM observations of AgNW-1; (**d**) TEM observations and (**e**,**f**) SEM observations of AgNW-2. The average length and diameter for AgNW-1 (from CEA-LITEN) were around 8 µm and 70 nm, respectively, and for AgNW-2 (from R&Dnano), they were around 119 nm and 11 µm, respectively.

In that context, we first investigated the influence of synthesis conditions on morphology and optical transmittance of AgNWs. AgNW-1 and AgNW-2 (from CEA-LITEN and R&Dnano, respectively) were both synthesized by a polyol process with different synthesis conditions, as described in Section 2.1. Figure 1 exhibits TEM and SEM observations of these AgNWs. 

**Figure 2:**
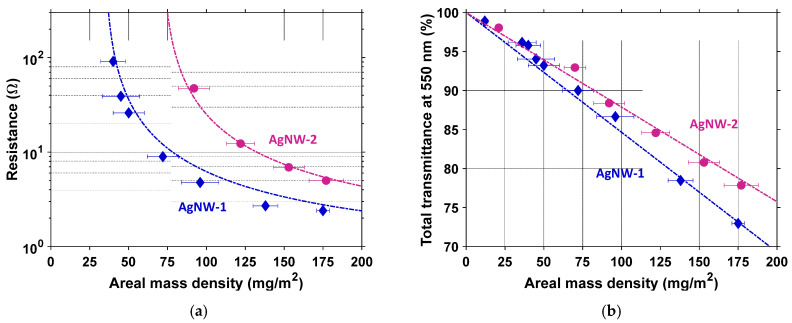
(**a**) Dependency of resistance, *R*, of the AgNW network versus *amd*; (**b**) Dependency of the total transmittance, *T_r_*, of the AgNW network versus *amd*. Symbols (blue diamond and pink circle) correspond to experimental data of AgNWs (AgNW-1 and AgNW-2, respectively), while dashed lines correspond to *R* and *T_r_* fitting using Equation (2) and Equation (3), respectively. The substrate contribution was removed.

One could compare the thickness of the PVP layer for both AgNWs observed using TEM (Figure 1a,d). The PVP layer of AgNW-1 of around 2 nm was thinner than the one of AgNW-2 of around 5 nm. SEM pictures revealed that their morphologies were different: AgNW-2 appeared with sharper features (Figure 1e) compared to AgNW-1 (Figure 1b). Moreover, one could observe the presence of silver particles with AgNW-2 (Figure 1f) compared to AgNW-1 (Figure 1c). Silver particles were not completely removed by centrifugation during AgNW synthesis. Figure 2 reports the influence of the *amd* of the AgNW network on electrical resistance, *R*, (Figure 2a) and total transmittance, *T_r_* (Figure 2b). Figure 2 exhibits experimental data for AgNW-1 and AgNW-2 after thermal annealing. Details about their optimization are provided in Section 3.2.2. The fitting of the experimental data was performed by Equation (2) and Equation (3) for *R* and *T_r_*, respectively. This clearly showed a good agreement with the experimental observations and validated the models that link the physical properties with *amd* and AgNW morphologies. The *amd_c_* for AgNW-1 and AgNW-2 were 35.6 mg/m² and 71.6 mg/m², respectively. AgNW-1 networks were more conductive at the same *amd*, due to a lower *amd_c_* and less of a PVP layer, while the total transmittance was lower for the same *amd* due to the smaller diameter. 

#### 3.2.2. Effects of Thermal Treatment on Electrical Conductivity 

Many studies have reported that after deposition of AgNWs, the nanowire–nanowire contact resistance can still be high, due to the lack of intimate contacts and the potential presence of the PVP layer or organic residues. Ding et al. recently summarized post-deposition treatments used for reducing this contact resistance, including thermal treatment, capillary-forced induced cold-welding or mechanical pressing [60]. These techniques can drastically reduce the electrical resistance of the network. 

**Figure 3:**
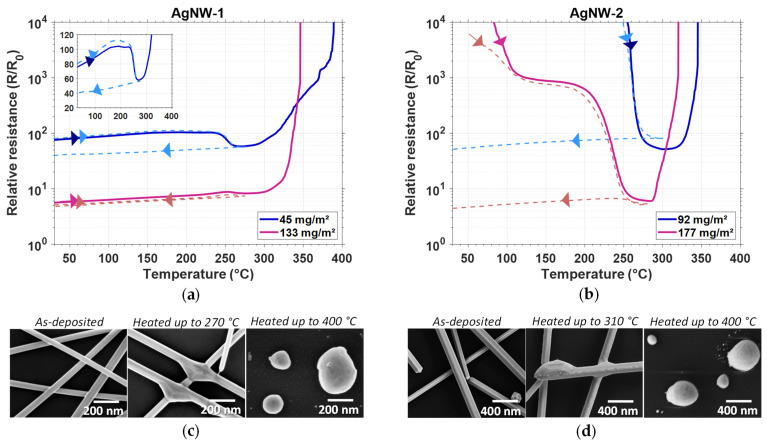
Effects of thermal treatment on AgNW’s electrical and structural properties for AgNW-1 and AgNW-2. Evolution in air of in situ electrical resistance of AgNW networks during a thermal ramp of 5 °C/min (solid line) (**a**) for AgNW-1 and (**b**) AgNW-2. Another specimen with a similar *amd* was thermally treated with the same thermal ramp (up to the temperature associated with the minimum electrical resistance (i.e., 270 °C and 310 °C for AgNW-1 and AgNW-2, respectively) and then cooled down to room temperature (dashed curve). The insert shows the linear dependence of both in situ resistance and temperature, according to Equation (4). SEM observations show the morphological change before (as-deposited) and after thermal treatment up to different temperatures for (**c**) AgNW-1 and (**d**) AgNW-2.

In this part, we wanted to compare the response to thermal treatment of both studied AgNW types. As presented in Figure 3, three main steps could be observed during full thermal ramps (black solid lines) for both AgNW-1 and AgNW-2. First, the electrical resistance, *R*, increased linearly with temperature until 230 °C. Then, a decrease of *R* was observed, which could be associated with a local sintering mechanism (occurring at the junctions between AgNWs). Networks of AgNW-2 (Figure 3b) were not conductive before thermal annealing, probably due to a thicker PVP layer. Their electrical resistance only started to decrease, by several orders of magnitude, at temperatures higher than 200 °C (Figure 3b). According to previous studies [40,61], the removal of residual solvents and PVP degradation occur for temperatures lower than 250 °C. Both AgNW types exhibited a short resistance plateau associated with an optimum resistance, around 270 °C. The temperature at which the optimum resistance was reached was slightly lower for AgNW-1, very probably because they had a smaller diameter (79 nm) than the ones of AgNW-2 (119 nm). This observation matched well with the previous study of Lagrange et al. [37]. Moreover, this resistance plateau proved that most junctions had been well optimized, as was verified by SEM (middle images of Figure 3c,d), compared to non-sintered AgNWs (left images of Figure 3c,d). Indeed, once activated during thermal annealing, the surface atomic diffusion, driven by surface energy reduction, induced local sintering at the junctions at first, followed by morphological instability of the nanowires (right images of Figure 3c,d) with AgNWs spheroidization (i.e., Plateau–Rayleigh instability) [37]. These results can be observed on both silver and gold nanowires, as shown by the recent work of Vigonski et al. [62]. 

For each type of AgNW, networks with a similar *amd* were thermally annealed up to the optimum temperature (red dashed lines in Figure 3a,b). It is worth mentioning here that the effects of annealing for both types of AgNWs appeared well reproducible during our experiments (i.e., the curves, such as those reported in Figure 3a,b, associated with one type of AgNW were nearly identical). After this thermal treatment, the electrical resistance linearly decreased during cooling down (inserts in Figure 3a,b). This was due to the reversible variation of the metallic behavior of AgNW networks and its linear dependency with temperature, *T*, as follows:

(4) $$R\left(T_{0}+\Delta T\right)=R_{0}\left(T_{0}\right)\times \left(1+\beta \times \Delta T\right)$$

where β is the temperature coefficient of resistivity. The extrapolated coefficients exhibited very similar values for both types of AgNW: 2.3 × 10^−3^ K^−1^ and 2.2 × 10^−3^ K^−1^ for AgNW-1 and AgNW-2, respectively. This value was the same as the one observed by Lagrange et al. for another type of AgNW [37]. Furthermore, while the *amd* of AgNW-1 (45 ± 10 mg/m²) was about half of the one of AgNW-2 (92 ± 7 mg/m²), the resistance at room temperature after annealing was nearly similar: 40.5 Ω and 47.3 Ω, and the optical transmittance at 550 nm was 94% and 88.5% for AgNW-1 and AgNW-2, respectively. We suspect that the difference in the behavior of electrical resistance under thermal stress depending not only on AgNW dimensions, but also on the AgNW synthesis method, especially for the AgNW-1 and AgNW-2. Indeed it originates from differences in the AgNW surface chemistry which depends, for instance, upon the thickness of the PVP layer.

Several studies, including ours, demonstrated that thermal treatment is an efficient post-deposition treatment to optimize the electrical resistance of an AgNW network in a reproducible way. If the thermal ramp is not limited to an optimum temperature, it leads eventually to a sharp increase of the electrical resistance due to the irreversible degradation of the AgNWs. 

**Figure 4:**
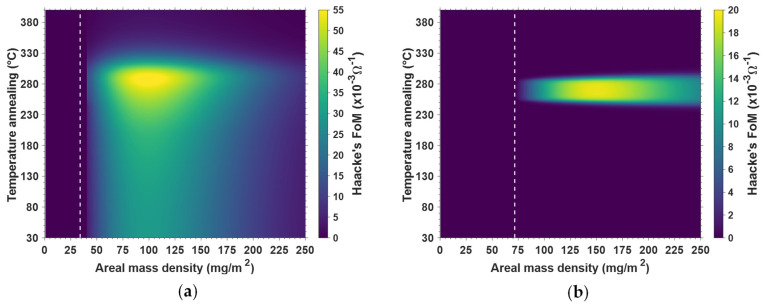
Combined effects of thermal treatment (through the maximum temperature of thermal ramp) and AgNW network density (*amd*) on the *FoM^Haacke^* of AgNW networks for (**a**) AgNW-1 and (**b**) AgNW-2. The vertical white dashed lines correspond to the critical *amd*, below which no percolation is generally observed.

The next section of the present study is to analyze the correlation between *amd* and the annealing temperature, in order to explore the influence of both *amd* and thermal annealing on the properties’ optimization, for both types of AgNWs. Figure 4 exhibits the iso-values of the *FoM^Haacke^*, as defined by Equation (1), plotted in the diagram, where the y-axis is the maximum annealing temperature (during a thermal ramp at 5 °C/min), and the x-axis is AgNW network *amd*. Figure 4a,b correspond to AgNW-1 and AgNW-2, respectively. These figures were obtained from data similar to those reported in Figure 3a,b but with several *amd* values for each AgNW type. The data *R*(*T*) was then fitted, for different *amd* and maximum temperature values, and the *FoM* values were calculated thanks to Equations (1)–(3). The vertical white dashed lines correspond to the critical *amd*, below which no percolation is generally observed [63]. 

Figure 4a,b also show the typical annealing temperature and *amd* ranges within which AgNW networks can be optimized. The prevailing difference between Figure 4a,b ws that AgNW-1 type, even without any thermal annealing, could reach *FoM* values of about 20 × 10^−3^ Ω^−1^, while thermal annealing was clearly mandatory to get a reasonably low resistance for AgNW-2. The origin of such differences very probably stems from differences in the surface chemistry between these two types of AgNWs. Actually, the surface chemistry that should be considered is related to several parameters that directly depend upon AgNW synthesis conditions and any post-deposition treatment. These parameters are, for instance, the thickness of the PVP layer, chemical state of PVP that could degrade following thermal treatment in air, precise shape and roughness of AgNW surfaces and any chemical products that can still be adsorbed at the surfaces of AgNWs in addition to PVP. Another example of such a difference between AgNW types has already been discussed by Madeira et al. [64]. This clearly shows the importance of the source of AgNWs, and one cannot deduce universal conditions for optimizing AgNW network properties, since precise conditions for optimizing AgNWs appear to depend on the origin of the AgNWs. 

### 3.3. Influence of Thin Oxide Coating on AgNW Network Stability

While AgNW networks appear very promising for many applications, their chemical, thermal and electrical instabilities constitute crucial drawbacks for their efficient integration as transparent electrodes within devices [43,65]. In our previous studies, oxide coatings, including zinc oxide (ZnO) [32,46], alumina (Al_2_O_3_) [66] and aluminum-doped zinc oxide (AZO) [67], deposited by AP-SALD were investigated to protect AgNW networks. For instance, a recent work showed that in addition to limited Ag mobility, the oxide coating provides a stabilizing effect thanks to a better homogenization of the current density across the sample [32]. The main advantage of the AP-SALD deposition technique is that it is a low-temperature process, as with the traditional Atomic Layer Deposition (ALD) technique, but with the additional advantages of being easily up-scalable, low-cost, vacuum-free and faster [33]. Recently, some SnO_2_/AgNW nanocomposites were investigated for TE [68,69], but none of them have been obtained by AP-SALD. Moreover, SnO_2_ appears to be a very common transparent oxide, specifically used, for instance, in photovoltaics when doped with fluorine [70]. This study presents a proof of concept about thermal stability enhancement of AgNW networks coated with SnO_2_ by AP-SALD. The SnO_2_/AgNW nanocomposites are compared to ZnO/AgNW nanocomposites, which could be considered as a reference, since they have already been investigated, for instance, by Khan et al. [46].

**Figure 5:**
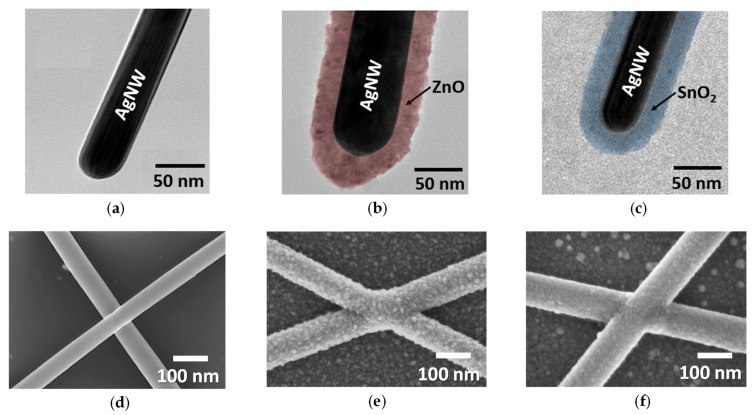
Coating AgNWs with thin oxide films by AP-SALD. TEM observations of (**a**) bare AgNW; (**b**) ZnO/AgNW and (**c**) SnO_2_/AgNW nanocomposites. SEM observations of (**d**) bare AgNW; (**e**) ZnO/AgNW and (**f**) SnO_2_/AgNW nanocomposites. The ZnO and SnO_2_ coating thicknesses around the AgNWs were about 25 nm.

Figure 5a–c show TEM images of bare AgNW, ZnO/AgNW and SnO_2_/AgNW nanocomposites, respectively. It is worth noting that the coatings on AgNW were highly conformal for both ZnO and SnO_2_. The thickness of the ZnO and SnO_2_ coatings around the AgNWs was about 25 nm. Figure 5d–f exhibit SEM images of the junctions of bare AgNW, ZnO/AgNW and SnO_2_/AgNW nanocomposites, respectively. These SEM pictures clearly confirm that both oxides were conformably coated on top of the AgNWs and the substrate. The AgNWs used in Figure 5 were of the AgNW-1 type.

**Figure 6:**
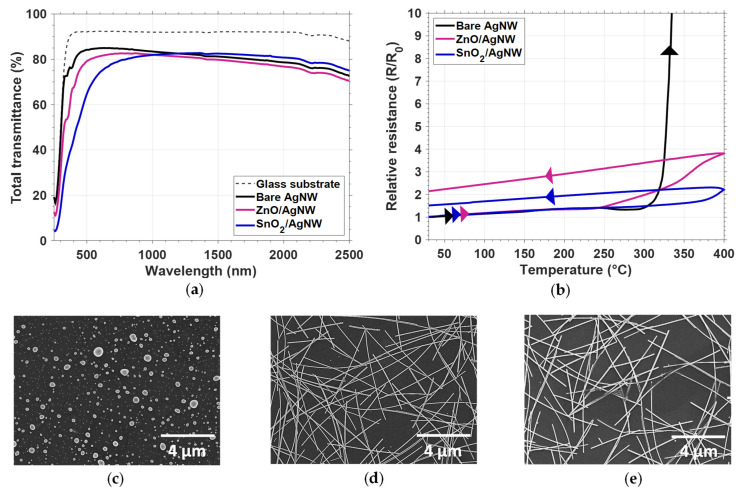
Effects of a thin oxide layer coating on optical transmittance and thermal stability for AgNW networks observed with a thermal ramp in air. (**a**) Dependency of total transmittance versus wavelength between 250 and 2500 nm for bare glass (grey dashed line), bare AgNW network (black continuous line), ZnO/AgNW (pink solid line) and SnO_2_/AgNW (blue solid line) nanocomposites; (**b**) Evolution of in situ electrical resistance of bare AgNWs (black solid line), ZnO/AgNW (pink solid line) and SnO_2_/AgNW (blue solid line) nanocomposites during a thermal ramp of 5 °C/min, from room temperature to 400 °C. SEM observations after a thermal ramp up to 400 °C of (**c**) bare AgNW networks; (**d**) ZnO/AgNW and (**e**) SnO_2_/AgNW nanocomposites. The ZnO and SnO_2_ coating around the AgNWs was about 25 nm thick.

The effects of oxide coating on optical transmittance and thermal stability of AgNWs were investigated. Figure 6a reports the total transmittance versus wavelength in visible and NIR regions for bare glass, bare AgNW networks, and ZnO– and SnO_2_– coated AgNW networks. Both ZnO and SnO_2_ coating were undoped, since no finite sheet resistance was measured using a 4-point probes measurement. At least in the visible range, the ZnO coating on AgNWs exhibited a slightly increased absorption compared to the bare AgNW networks, while the SnO_2_ coating appeared more detrimental. This was due the larger optical absorption coefficient of undoped SnO_2_ (α = 1.46 × 10^4^ cm^−1^) compared to ZnO (α = 10^3^ cm^−1^) [71]. However, in the NIR region, the antireflection coating associated with the SnO_2_ layer increased the transmittance. For photovoltaic applications, for example, using a thinner SnO_2_ layer would appear more attractive. 

The resistance of bare AgNWs, ZnO/AgNWs and SnO_2_/AgNWs was 12.7 Ω, 12.0 Ω and 13.3 Ω, respectively. Figure 6b exhibits the relative resistance of these three AgNW-based nanocomposites during a thermal ramp with a rate of 5 °C/min. As discussed in detail in Section 3.2.1, bare AgNWs (black line) first showed a linear increase in electrical resistance, followed by a hump, due to a local sintering mechanism. Then, the electrical resistance sharply increased due to the AgNW morphological (Plateau–Rayleigh) instability, caused by the thermal activation of silver atomic surface diffusion. The spheroidization of the bare AgNW after a thermal ramp up to 400 °C can be observed in the SEM image of Figure 6c. Compared to bare AgNW networks, ZnO/AgNW and SnO_2_/AgNW networks were still highly conductive after a full thermal ramp up to 400 °C (Figure 6b). The presence of these metal oxide coatings efficiently prevents the surface atomic diffusion of silver atoms at the origin of the electrical properties’ degradation. The good morphological stability of the ZnO/AgNW and SnO_2_/AgNW network after a thermal ramp up to 400 °C is depicted in the SEM images of Figure 6d,e. No signs of degradation could be observed for the majority of AgNWs coated by ZnO or SnO_2_. Only some AgNWs were partially degraded, and this could explain the low relative resistance change after thermal stress. 

As with the ZnO coating, this study demonstrated that even a thin SnO_2_ coating deposited by AP–SALD drastically enhances the thermal stability of AgNW networks. Further studies are required to better investigate the impact of SnO_2_ thickness on the physical properties of the AgNW networks, as well as their adhesion to the substrate and the enhancement of their electrical and ageing stability. 

## 4. Conclusions and Prospects

The present article has discussed different features of AgNWs and AgNW networks that drastically influence the properties of AgNW networks. We highlighted the significance of key parameters, such as the AgNW synthesis method, network density, post-deposition treatments and the coating of AgNWs by thin oxide layers. All of these parameters should be carefully chosen during the optimization of such a transparent electrode, taking into account the targeted application.

Several studies, including ours, have demonstrated that thermal annealing is an efficient post-deposition treatment to optimize the electrical resistance of AgNW networks in a reproducible way. However, if the thermal annealing is not limited to an optimum temperature, it leads to a sharp increase of the electrical resistance, due to irreversible degradation of the AgNWs. We showed that the behavior of electrical resistance under thermal stress depends on the synthesis method of AgNWs and, especially, on the surface chemistry of the AgNWs (linked, for instance, with the PVP layer thickness). This point has not been investigated much in literature so far and would certainly deserve to be explored in depth. To find the optimal treatment conditions, we investigated a novel way, which went beyond traditional *FoM*, and related it with the annealing temperature.

The morphological stability of AgNW networks is crucial for their performance. To address this, great attention has been devoted lately to the effects of coatings in order to enhance stability under thermal stress. This study demonstrated that even thin SnO_2_ coatings deposited by AP-SALD drastically enhance the thermal stability of AgNW networks. Further studies are required to better investigate the impact of SnO_2_ thickness on the physical properties of AgNW networks, as well as the enhancement of their electrical, thermal and chemical stability. Moreover, SnO_2_ appears to be a very common transparent oxide, specifically used in photovoltaics when doped with fluorine. One can clearly anticipate that numerous research studies will be devoted to coating AgNW networks with different materials, most likely to target emerging applications that have not yet been intensively considered for AgNW networks, such as low-emissivity or antimicrobial applications. 

Finally, we can assume that all efforts devoted to AgNWs will also be applied to other types of metallic nanowires, such as core-shell or bimetallic nanowires, which can demonstrate novel and complementary properties for efficient integration in industrial devices.

## Figures and Tables

**Table 1:** Synthesis conditions and dimensions (Length, L, and Diameter, D) of two types of AgNWs.

Name	Suppliers	Details of the Synthesis	Dimensions of AgNWs
AgNW-1	CEA-LITEN (France)	(1) Stirring of a 160 mL ethyleneglycol (EG) solution with NaCl (1 mM) and PVP (3.54 g, M_w_ = 40,000 g/mol) at 120 °C and cooling down to room temperature (2) Addition of this solution into 80 mL of an EG solution of AgNO_3_ (1.36 g, 0.1 M) at 120 °C and further heated at 160 °C, refluxed for 80 min and cooled down to room temperature (3) Washing of the remaining grey residue containing nanowires after two settlement days with acetone and dispersion of the AgNWs in methanol	L = 8 ± 3 µm D = 79 ± 10 nm
AgNW-2	R&Dnano (France)	(1) Stirring of an EG solution with NaCl (5.5 µM), PVP (72 mM) and AgNO_3_ (59 mM) at room temperature (2) Heating of the solution at 130 °C for 5 h (3) Washing steps (three steps with water and one with isopropanol), followed by a centrifugation step	L = 11 ± 5.8 µm D = 119 ± 23 nm

**Table 2:** Overview of the main properties of AgNW networks and recent ways to optimize these physical properties.

Associated Network Properties	Ways to Optimize These Properties	References
Optical transparency and electrical resistance (evaluated by $FoM^{Haacke}$)	The denser the network, the less transparent, more conductive and hazier the transparent electrode	[36,37]
	Distribution of the AgNW network: spray-coating deposition leads to a higher electrical homogeneity than spin coating	[38]
	Post-deposition treatments can drastically decrease electrical resistance	[39,40,41,42]
	Different synthesis conditions of AgNWs have an influence on not only optical transparency and electrical resistance, but also on response to thermal treatment of the AgNW network	Present work
Morphological stability	AgNWs with small diameters degrade faster, and AgNW networks with higher densities appear more stable	[37,43]
	Ag-based bimetallic NWs exhibit stronger oxidation resistance compared to AgNWs	[44]
	Development of nanocomposites by coating AgNW with an oxide layer to enhance thermal and electrical stability	[45,46] Present work
Haziness	Small AgNW diameter or ultra-long nanowires lead to lower haziness	[45,47]
Surface roughness	Post-deposition treatments can be performed to diminish the surface roughness of the network	[42,48]
	AgNWs can be embedded into a polymeric matrix to diminish the surface roughness of networks	[49,50]
Adhesion to substrate	Coating of AgNWs by a thin oxide layer or the use of self-adhesive coating ink or nanocelluloses to improve adhesion of AgNWs to the substrate	[22,51,52]

## Data Availability

The data presented in this study are available on request from the corresponding author.
